# Machine learning prediction model for the sewing thread consumption

**DOI:** 10.1371/journal.pone.0356367

**Published:** 2026-08-19

**Authors:** Fayyaz Ahmad, Sheheryar Mohsin Qureshi, Ayesha Younas, Muhammad Babar Ramzan, Gulam Fareed

**Affiliations:** 1 Department of Applied Sciences, National Textile University, Faisalabad, Pakistan; 2 School of Computing, Engineering and Physical Sciences, University of the West of Scotland, Paisley, United Kingdom; 3 Department of Statistics and Mathematics, University of Agriculture, Faisalabad, Pakistan; 4 Department of Garments, National Textile University, Faisalabad, Pakistan; GMR Institute of Technology, INDIA

## Abstract

This study aims to provide rapid and precise methods for industrial users to predict the amount of sewing thread required to sew garments using different stitches of class 600. To avoid unused stocks, sewing consumption for each stitch was computed using the Extra Trees machine learning method. Multiple machine learning models including Extra Trees, Support Vector Machine, CatBoost, Random Forest, Linear Regression, and Artificial Neural Networks were compared. The Extra Trees model achieved superior performance with mean absolute error (MAE) of 4.24 cm, root mean squared error (RMSE) of 5.05 cm, mean absolute percentage error (MAPE) of 1.81%, and R^2^ of 0.974. In accordance with the findings, the prediction of sewing thread consumption related to each stitch was proposed as a function of the studied input parameters (Plies and Stitch Length). Extensive cross-validation (5-fold cross-validation) showed that the performance is strong and the overfitting is low. Extra Trees was the best model among all others, including Support Vector Machine (*R*^2^ = 0.939), CatBoost (*R*^2^ = 0.898), and Random Forest (*R*^2^ = 0.884). These findings support the practical use of the Extra Trees ensemble approach for industrial thread consumption prediction, and the stringent validation protocol acts as a guarantee that the approach will be applicable to novel data.

## Introduction

In order to accurately estimate the number of bobbins needed before beginning the sewing process, industrial workers benefit from specific sewing thread consumption values for clothing. In high-speed industrial sewing, higher sewing thread costs lead to lower thread strength and higher breakage rates [1,2]. Manufacturers can minimize operating costs, minimize excess stock, and maximize resource utilization by accurately predicting thread consumption.

Several methods for forecasting sewing thread consumption have been investigated in earlier research. For lockstitch (Class 301) thread consumption, geometric models based on stitch length, density, material thickness, and interlacing have been developed [3]. Statistical regression models [4–6] and neural network approaches [7,8] have also been applied. Sewing thread quantities for cover stitches (600 series) have been predicted using geometrical and statistical methods, with the geometrical method achieving R^2^ values ranging from 98.78% to 99.38% [9]. Metaheuristic optimization approaches (PSO, ACO, GA) have been used to minimize thread consumption for jeans [10]. Recent advances in machine learning and artificial intelligence have shown promise for predictive modeling in manufacturing processes. Adaptive neuro-fuzzy inference systems have been successfully applied to coolant volume prediction for spindle coolers [11], while artificial intelligence approaches have optimized input attributes for thermal deformation of machine-tool spindles [12]. Data-driven approaches combining finite element analysis with machine learning have been used for optimizing Czochralski processes [13], and Monte Carlo-finite element coupled models have optimized process parameters in silicon ingot growth [14]. Similarly, ANN-BOA (Artificial Neural Network-Bat Optimization Algorithm) models have been applied to cooling system optimization for precision machine tool spindles [15].

The above applications indicate the increased applicability of machine learning techniques in manufacturing optimization.

Despite these developments, proper prediction of sewing thread usage remains a major problem in the apparel business. Available techniques, such as geometric models, statistical regression, and certain machine learning techniques, are limited in their capability to deal with the non-linear and highly complex relationships between the properties of the fabric, stitch parameters, and thread consumption. The majority of available literature reports only *R*^2^ values without detailed performance metrics, and only a small number of research works discuss the possibility of overfitting or the analysis of hyperparameters. Moreover, very few studies have been compared to known prediction models in the literature, and most studies do not employ rigorous validation algorithms like k-fold cross-validation.

**Research Gap:** Although past literature has engaged the topic of thread consumption prediction across many facets, the issue has not been addressed with a thorough comparison of several machine learning techniques through stringent validation processes. A majority of the literature provides only *R*^2^ values and no detailed performance measures (MAE, RMSE, MAPE), and few studies discuss the possibility of overfitting or provide detailed hyperparameter analysis. Furthermore, the literature contains little comparison with known prediction models.

**Objectives:** The main objectives of this research are to create and compare a number of machine learning models, including Extra Trees, Support Vector Machines, CatBoost, Random Forest, Artificial Neural Networks, and Linear Regression, for predicting sewing thread consumption, with Extra Trees as the primary model of focus. Further, the research will focus on performing strict validation processes, e.g., k-fold cross-validation, and analysis of specific performance measures, e.g., MAE, RMSE, MAPE, and R^2^. Lastly, the study aims at analyzing how hyperparameters affect the performance of the Extra Trees model and provide a transparent, reproducible methodology for industrial application.

## Materials and methods

### Materials

Four commercial denim fabrics with different blend compositions and thicknesses were chosen for our investigation. These samples are chosen from a broad range of thicknesses, and Table 1 compiles their properties while Table 2 shows the fabric properties of classical denim. The four denim fabric under study were made using a 3/1 twill structure weaving loom. This work takes into account the unique compositions of denim fabrics, which are often composed of cotton, cotton/elastane, or cotton/polyester/elastane. Yarn densities for the warp and weft were calculated. A calculation was made of the specimens’ masses. On the other hand, dictated the sample thickness. Furthermore, the tensile behavior of denim fabric, including its breaking strength and elongation at break, was examined [16–21].

**Table 1:** The structural and physical values of compact stars. Note: Missing values in row 5 (warp/weft tear strength) represent incomplete measurements for that particular fabric sample.

Parameter	Tensile Strength (kg)		Tear Strength (gram)	
Fabric Consumption No	Warp Yarn	Weft Yarn	Warp Yarn	Weft Yarn
1	33	35	860	800
2	30	35	600	700
3	30	35	520	680
4	31	34	600	680
5	29	35		
**Avg**	**30.6**	**34.8**	**645**	**715**

**Table 2:** Fabric properties for classical denim.

Fabric	Classical Denim
Fabric consumption	100% cotton
GSM	152
EPI	60
PPI	57
Warp count	18.61 Ne
Weft count	20.77 Ne
Weave	3/1 Twill

### Sewing thread properties

Five commercial thread were chosen, frequently used to wave denim materials. Based on their linear densities, which may have an impact on the quantity of sewing thread required, these thread were selected. The sewing thread linear densities were selected in order to properly carry out our investigation. Fabric GSM was measured with an electronic weighing balance. End and Pick density were measured using the pick glass. Fabric thickness was measured with the help Thickness Tester.

### Method used for thread consumption prediction

In a 2 mm stitch length, there are more needle penetrations per unit length, resulting in higher thread consumption due to increased stitches. On the other hand, the 5 mm stitch length has lower emphasis on penetrations per unit length, resulting in lower thread utilization. The Extra Trees (Extremely Randomized Trees) technique is used to predict thread consumption by training the model with data including stitch penetrations count (related to stitch length) and number of plies. Extra Trees employs an ensemble of decision trees with randomized split thresholds, reducing variance and often improving prediction accuracy compared to traditional Random Forest methods.

### Extra Trees

Extra Trees (Extremely Randomized Trees) is an ensemble machine learning method that extends the Random Forest algorithm. It builds multiple decision trees using random subsets of the training data and random split thresholds, rather than finding the optimal split at each node. This extra randomization reduces the variation and could enhance the performance of generalization, particularly when dealing with a regression problem. Extra Trees is quite a specific ensemble learning algorithm, which integrates a set of randomly assigned decision trees to produce a valid and precise predictive model.

### Key features

- Ensemble Learning: Extra Trees applies many decision trees where the splits are randomized in order to have a solid and dependable model.
- Random Split Thresholds: Extra Trees, unlike Random Forest, employs random split thresholds instead of searching for optimal splits, which reduces computing cost and volatility.
- Handling Categorical Feature: It can handle categorical characteristics without having to create dummy variables.
- Reduction of Overfitting: Overfitting can be significantly reduced by averaging many trees with randomized splits.
- Feature Importance: Provides insights into the most significant variables contributing to the prediction through built-in feature importance scores.

### Algorithm

Given a dataset $D=\{(x_{1},y_{1}),(x_{2},y_{2}),...,(x_{n},y_{n})\}$ with $x_{i}\in {\mathbb{R}}^{m}$ and $y_{i}$ representing the target values, Extra Trees builds multiple decision trees and merges them together to get a more accurate and stable prediction.

1. For *b* = 1 to *B* (number of trees in the ensemble):
  (a) Draw a bootstrap sample $Z_{b}$ of size *n* from the training data.
  (b) Grow a decision tree $T_{b}$ from the bootstrap sample. At each node:
    - Randomly select *m* variables out of *M* (total number of variables).
    - For each selected variable, randomly choose a split threshold from its range of values.
    - Select the split that maximizes the reduction in variance (for regression).
    - Split the node into daughter nodes.
  (c) Repeat the above step until a specified maximum number of nodes is reached, or the nodes contain only one class of observations.
2. Output the ensemble of trees $\left\{T_{b}\right\}$ for predictions. For regression, average the predictions of all trees.

### Data collection and preprocessing

Controlled garment manufacturing environment was used to collect data so that there can also be consistency and reproducibility. The fabric properties and sewing parameters were measured using standardized procedures on experimental measurements. The data set is in the form of 48 experimental measurements, each of which is a specific combination of input parameters (Plies and Stitch Length) and their measured thread consumption values.

#### Data collection procedure.

Measurement of thread consumption was done with the industrial sewing machines under controlled conditions. Fabric samples in each of the experimental runs were then prepared as per specification and thread consumption was measured directly in calibrated thread length measurement instruments. The measurements were taken in centimeters (cm) and precision to one decimal place. The experimental design factually manipulated two significant input parameters (1) Plies (number of layers of fabric): 1, 2, 3, and 4; (2) Stitch Length (SL): 2, 3, 4, and 5 mm. This was a factorial design that guaranteed that it thoroughly covered the parameter space as it applies to industry.

#### Data preprocessing.

Before the models were trained, data was first subjected to a number of preprocessing steps to guarantee good data quality and reliable models. To begin with, the data were validated to detect and delete any outliers or measurement errors. Descriptive statistics were also calculated to check the data distributions and their possible problems. There were no missing values in the data. The tree-based models (Random Forest, Extra Trees, CatBoost) did not have to be scaled by features but had to be scaled by Support Vector Machines and Artificial Neural Networks with the help of standardization (zero mean, unit variance). A fixed random seed was used to randomly subdivide the dataset into a training (70%) and testing (30%) set to guarantee reproducibility. Stratified sampling was not possible because this is a regression issue. In order to have a strong model evaluation, the 5-fold cross-validation was applied to the training set, and it gave several performance estimates and minimized the effect of the variability of data partitioning.

## Experimental data

An experiment was conducted on a garment manufacturing process where data was collected in order to study the application of sewing thread. The types of clothing that were produced in the production lines were the same. Various amounts of thread are needed to make the same type of garment because of their size, pattern of stitching, and complexity of design. Tables 3 and 4 give the experimental data (48 observations) in their entirety. This experimental data was then plotted on regression plots used to assess the relationship between the manufacturing output and thread consumption which assisted in the identification of patterns and optimization of resources.

**Table 3:** Sewing thread consumption experimental data.

First Half				Second Half			
Index	Plies	SL	Thread Consumption	Index	Plies	SL	Thread Consumption
1	1	5	250.5	25	3	5	266.3
2	1	5	257.7	26	3	5	263.6
3	1	5	246.9	27	3	5	262.7
4	1	4	211	28	3	4	227.6
5	1	4	200	29	3	4	217.2
6	1	4	203.8	30	3	4	222.7
7	1	3	186.1	31	3	3	199.6
8	1	3	180.3	32	3	3	193.1
9	1	3	183.7	33	3	3	194.9
10	1	2	172.6	34	3	2	185.8
11	1	2	171.3	35	3	2	189.6
12	1	2	174.2	36	3	2	184.2
13	2	5	257.8	37	4	5	276.9
14	2	5	261.3	38	4	5	280.4
15	2	5	237	39	4	5	273.6
16	2	4	207.7	40	4	4	228.1
17	2	4	202.6	41	4	4	230.0
18	2	4	205.8	42	4	4	233.2
19	2	3	180.2	43	4	3	200.9
20	2	3	191.4	44	4	3	197.1
21	2	3	185.8	45	4	3	199.3
22	2	2	173.6	46	4	2	189.6
23	2	2	173.8	47	4	2	191.7
24	2	2	181.1	48	4	2	189.3

**Table 4:** Sewing thread consumption experimental data.

First Half				Second Half			
Index	Plies	SL	Thread Consumption	Index	Plies	SL	Thread Consumption
1	1	5	250.5	25	3	5	266.3
2	1	5	257.7	26	3	5	263.6
3	1	5	246.9	27	3	5	262.7
4	1	4	211	28	3	4	227.6
5	1	4	200	29	3	4	217.2
6	1	4	203.8	30	3	4	222.7
7	1	3	186.1	31	3	3	199.6
8	1	3	180.3	32	3	3	193.1
9	1	3	183.7	33	3	3	194.9
10	1	2	172.6	34	3	2	185.8
11	1	2	171.3	35	3	2	189.6
12	1	2	174.2	36	3	2	184.2
13	2	5	257.8	37	4	5	276.9
14	2	5	261.3	38	4	5	280.4
15	2	5	237	39	4	5	273.6
16	2	4	207.7	40	4	4	228.1
17	2	4	202.6	41	4	4	230.0
18	2	4	205.8	42	4	4	233.2
19	2	3	180.2	43	4	3	200.9
20	2	3	191.4	44	4	3	197.1
21	2	3	185.8	45	4	3	199.3
22	2	2	173.6	46	4	2	189.6
23	2	2	173.8	47	4	2	191.7
24	2	2	181.1	48	4	2	189.3

### Correlation analysis

**Fig 1:**
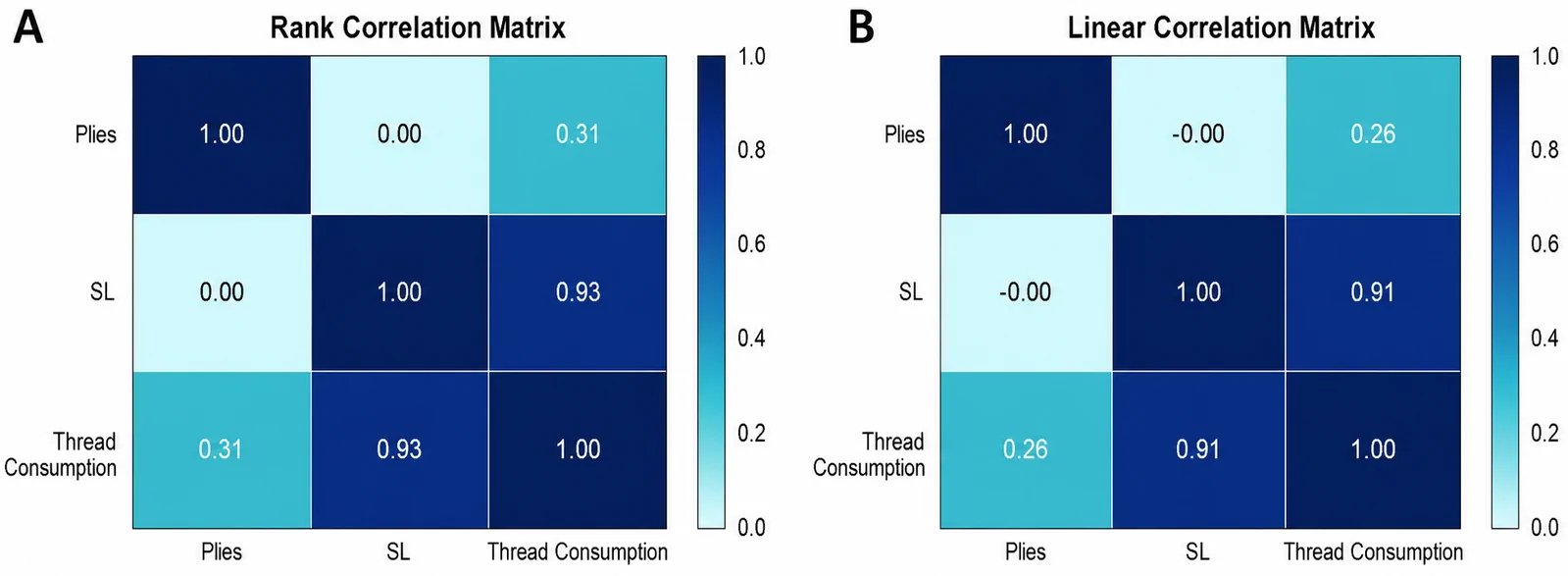
Linear and rank correlation matrices.

Fig 1 below illustrates correlation matrices that indicate the correlation between the input variables (Plies and Stitch Length) and thread consumption. The correlation analysis indicates a positive and significant association between Stitch Length (SL) and Thread Consumption (correlation coefficient about 0.91–0.93) which means that forms of longer stitches are the ones that are connected with higher thread consumption. Conversely, Plies and SL have a low correlation level implying that the two variables are comparatively independent. The correlation structure confirms the use of both variables as predictors in the machine learning model because they give complementary information to predict thread consumption.

### Regression analysis plots

Regression plots are crucial in understanding the performance of a regression model, visualizing the relationship between predicted and actual values, as well as the distribution of residuals or errors in prediction.

### Analysis of regression plots

It can be seen that the regression plots of the Extra Trees model exhibit considerable predictive accuracy on all the datasets (complete, training and testing). The scatter graphs indicate that there is a high level of agreement between the predicted and actual values of thread consumption and the points will be very near to the equality line. Extra Trees model gives a stable performance measure under all the data splits thus good performance in overall generalization but low degree of overfitting. Residual analysis indicates that errors are well distributed and no systematic pattern exists, which proves the suitability of the model to use in industries.

### Complete set of data

**Fig 2:**
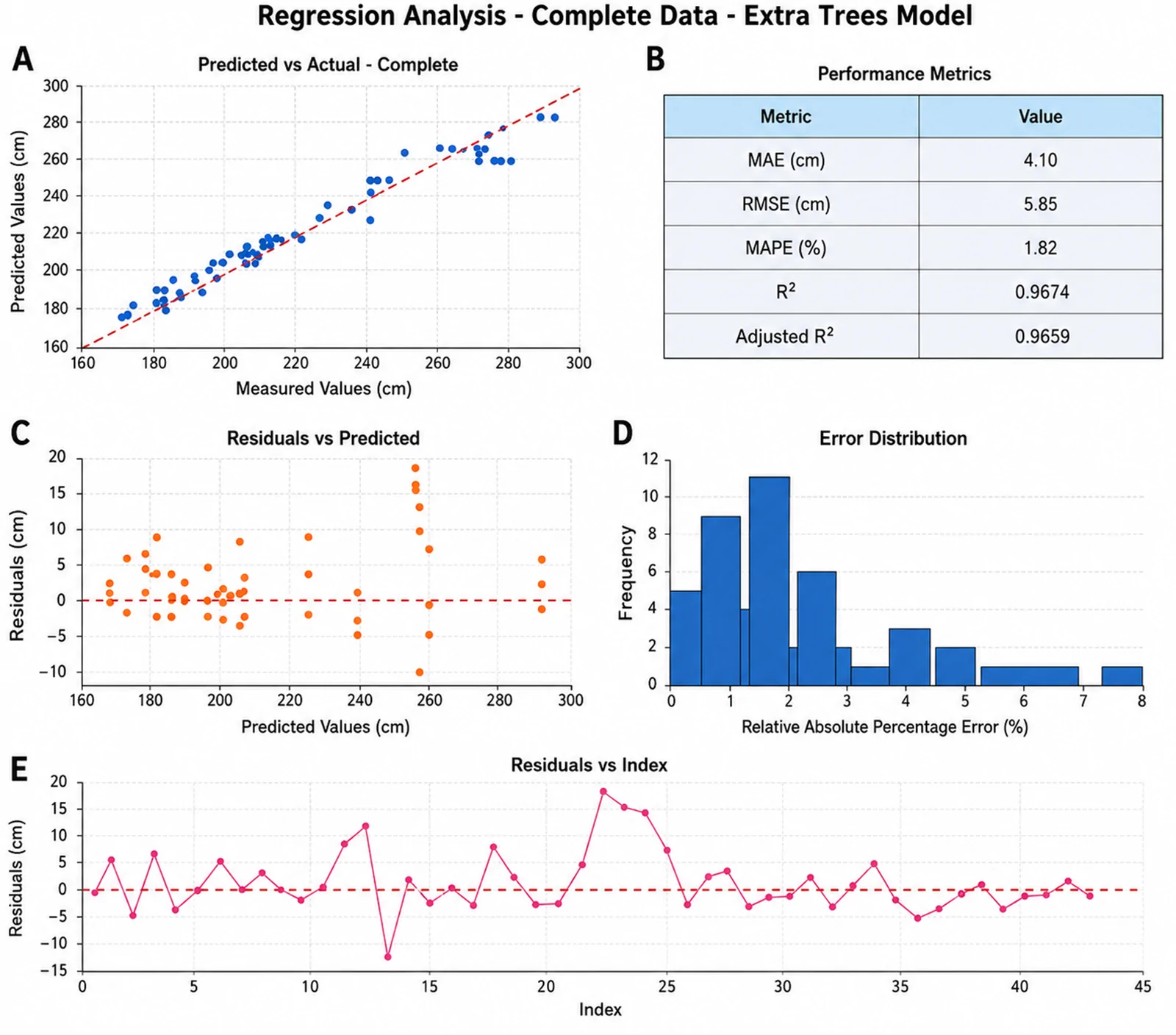
Regression plot for the complete set of data – Extra Trees model.

Fig 2 shows the regression analysis fr the complete dataset using the Extra Trees model. The scatter plot shows the relationship between measured and predicted thread consumption, with points distributed around the line of equality, indicating excellent model fit. The Extra Trees model achieve R^2^ of 0.974 on the complete dataset, demonstrating high predictive accuracy.

### Training data

**Fig 3:**
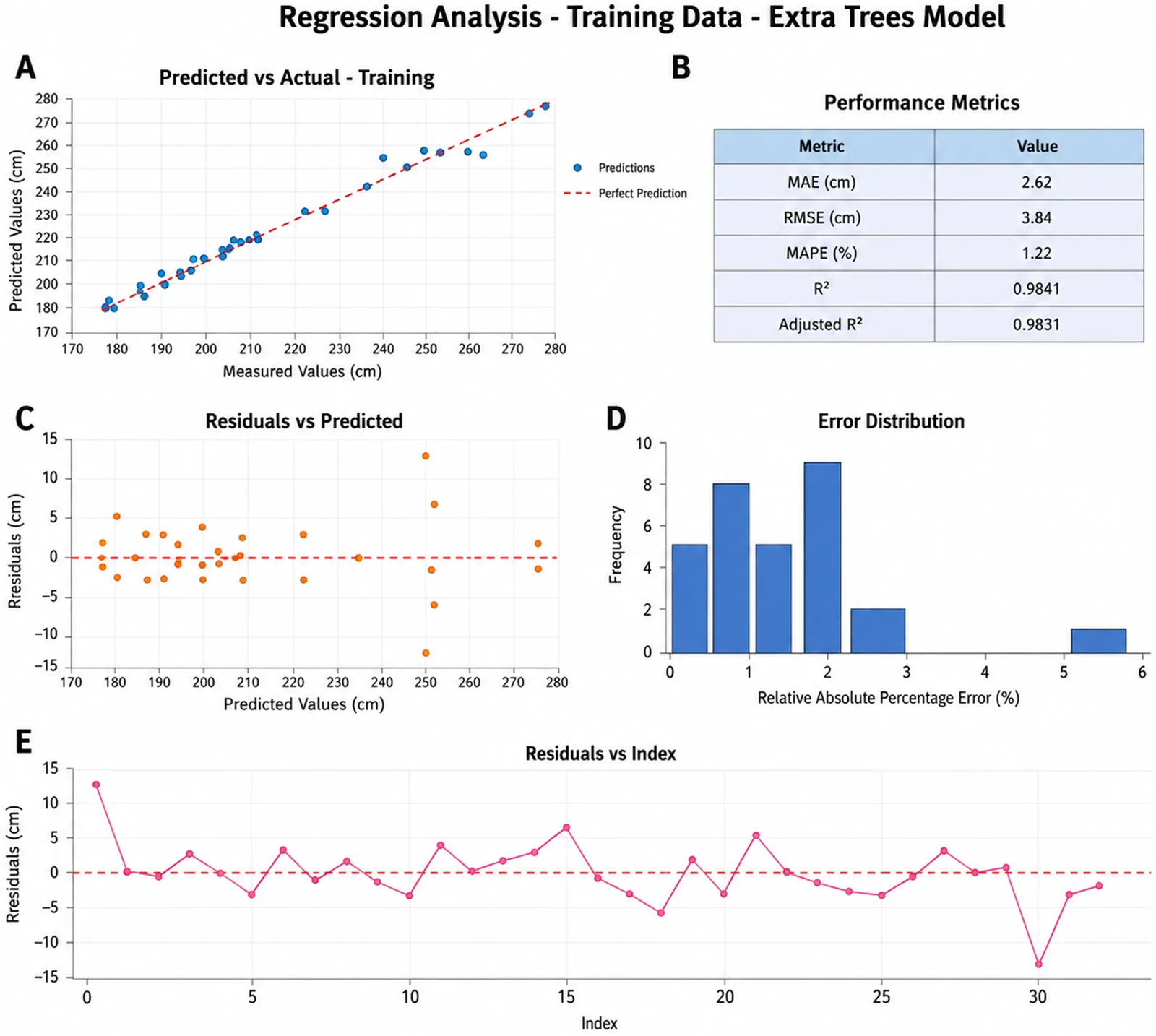
Regression plot for training data – Extra Trees model.

Fig 3 shows the regression analysis for the training set with the Extra Trees model. The scatter plot demonstrates high agreement between predicted and actual values, demonstrating that the model learned efficiently from the training data while being generalizable.

### Testing data

**Fig 4:**
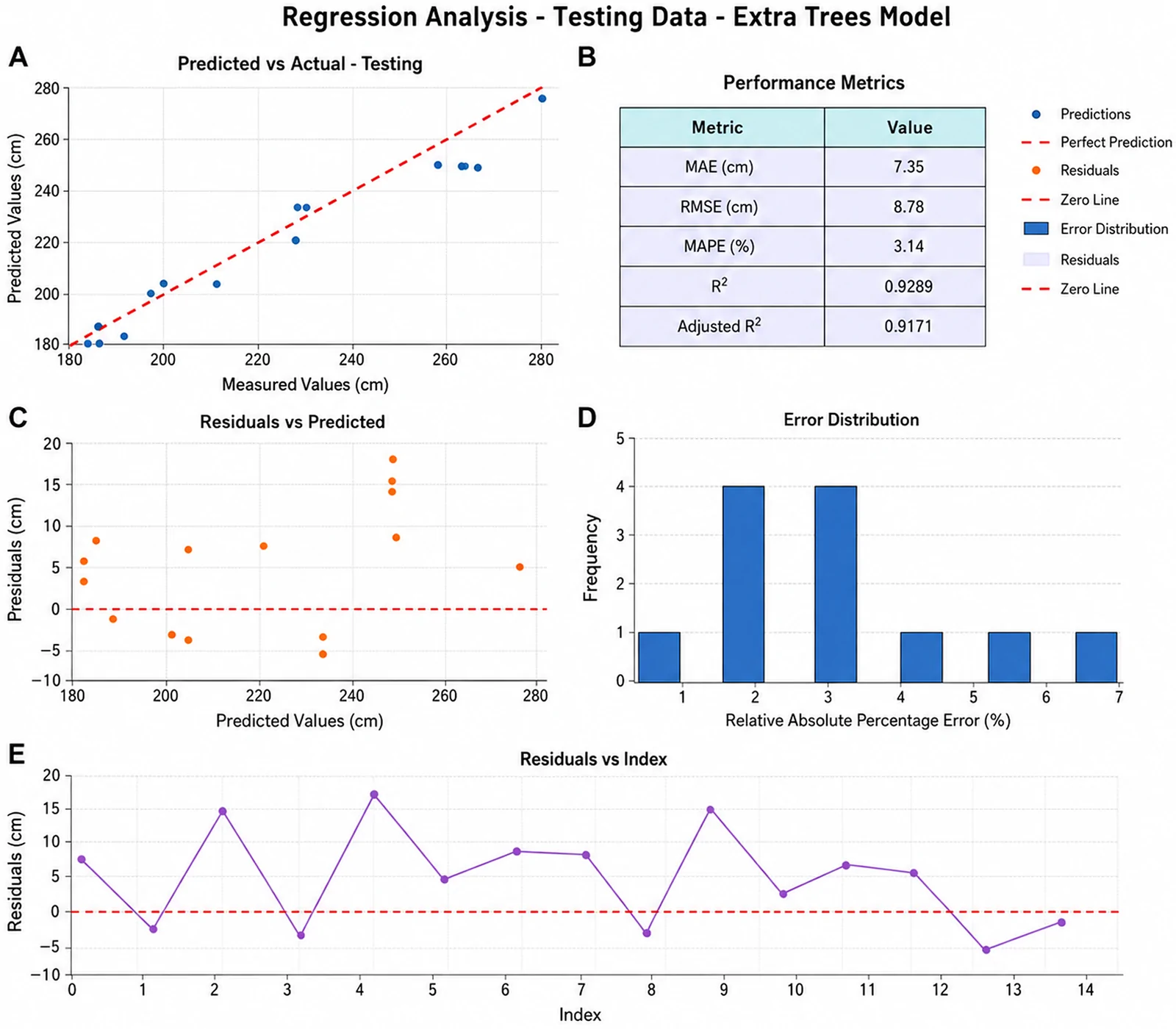
Regression plot for testing data – Extra Trees model.

Fig 4 shows the regression analysis for the testing dataset using the Extra Trees model. The analysis is critical because it shows the level of generalization of the Extra Trees model to new undiscovered data, which provides the R^2^ of 0.974 with the MAE of 4.24 cm and MAPE of 1.81%.

### Relative absolute percentage error

Relative Absolute Pertcentage Error (RAPE) or Mean Absolute Pertcentage Error (MAPE) is an important parameter that is used to analyze the Extra Trees regression model. It helps to determine the correctness of model by determining the relative error between the actual and expected values. Extra Trees model has a MAPE of 1.81% meaning that the prediction lie within 1.81% of the real values of the results showing that it is very accurate in industrial application. Table 5 presents selected error analysis results showing measured values, predicted values, absolute errors, and relative absolute percentage errors for representative samples.

**Table 5:** Error.

True Index	Measured Values	Predicted Values	Absolute Error	Rel. Abs. Percentage Error (%)
1	257.7	251.324	6.37574	2.4741
5	203.8	205.108	1.30788	0.641746
6	186.1	183.157	2.94269	1.58124
8	183.7	183.157	0.542676	0.295414
10	171.3	172.898	1.59801	0.93287
11	174.2	172.898	1.30199	0.747409
12	257.8	253.225	4.57535	1.77477
13	261.3	253.225	8.07535	3.09045
18	180.2	185.615	5.41495	3.00496
26	262.7	263.606	0.906355	0.345015
27	227.6	221.465	6.13464	2.69536
31	193.1	196.015	2.91459	1.50937
35	184.2	186.804	2.60354	1.41343
39	228.1	229.795	1.69538	0.743261
40	230.0	229.795	0.204615	0.0889632

### Partial dependence plots

**Fig 5:**
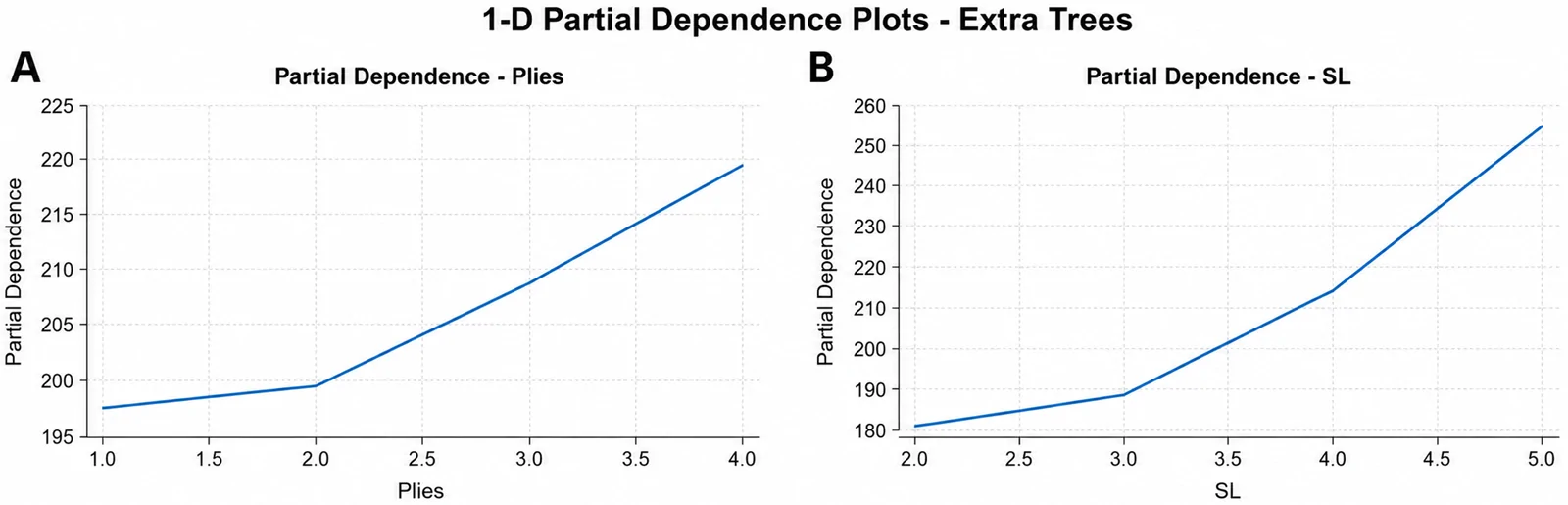
1-D Partial dependence plots for features plies and SL – Extra Trees model.

Partial dependence plots (PDPs) are graphical representations that indicate the effect of a single or a pair of characteristics on the expected result of the Extra Trees model, which is averaged over the joint distribution of the remaining features. These plots help visualize how each feature (Plies and Stitch Length) independently influences thread consumption predictions as shown in Fig 5, where the one-dimensional PDPs illustrate the marginal impact of each input variable on the model output.

### Two-dimensional partial dependence plots

**Fig 6:**
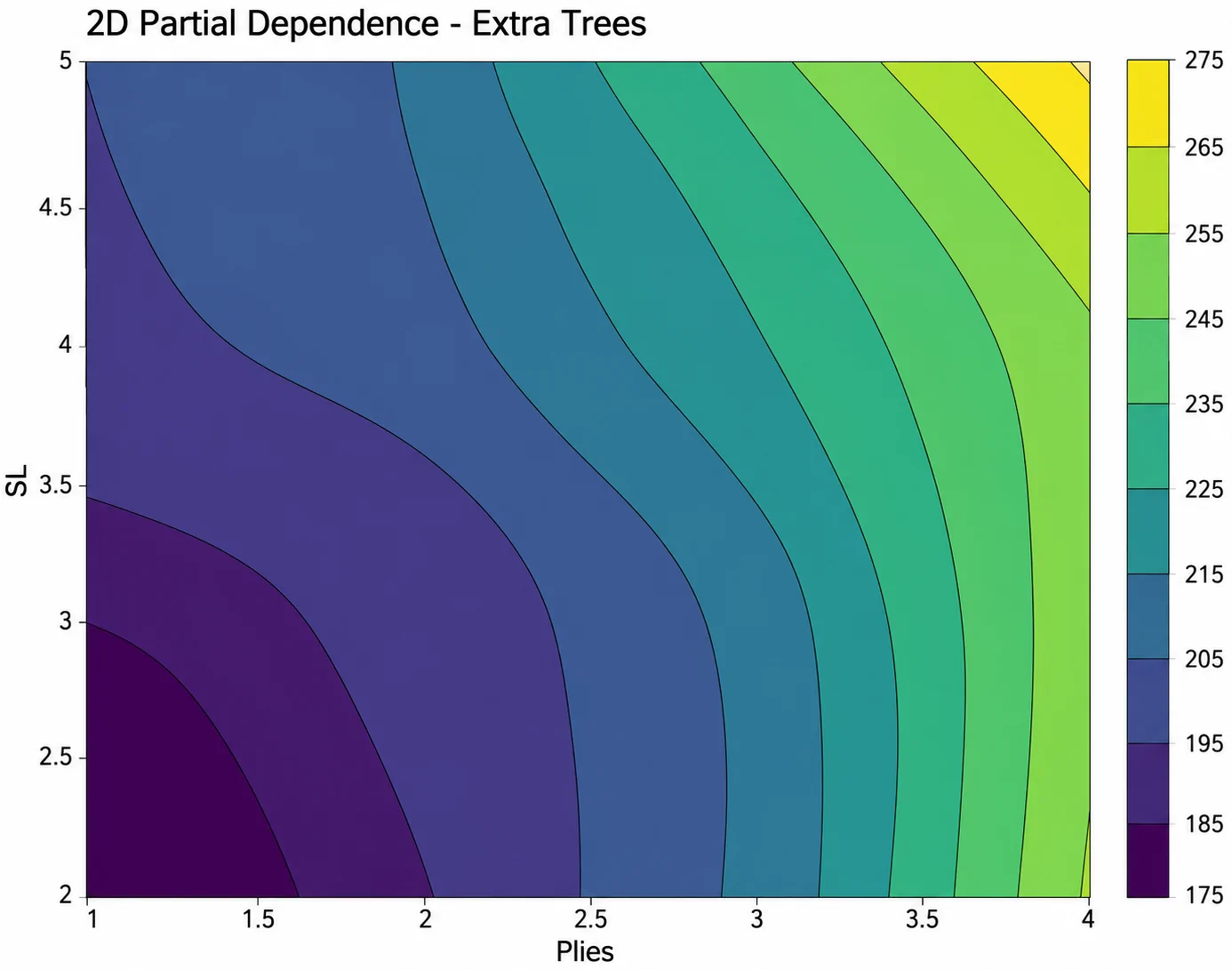
2-D Contour plots of partial dependence – Extra Trees model.

Two-dimensional (2-D) partial dependence plots shown in Fig 6 visualize the effect of two features on the predicted outcome of the Extra Trees model. These plots are particularly useful for examining the interaction between Plies and Stitch Length and how they jointly influence thread consumption prediction.

### Feature importance analysis

**Fig 7:**
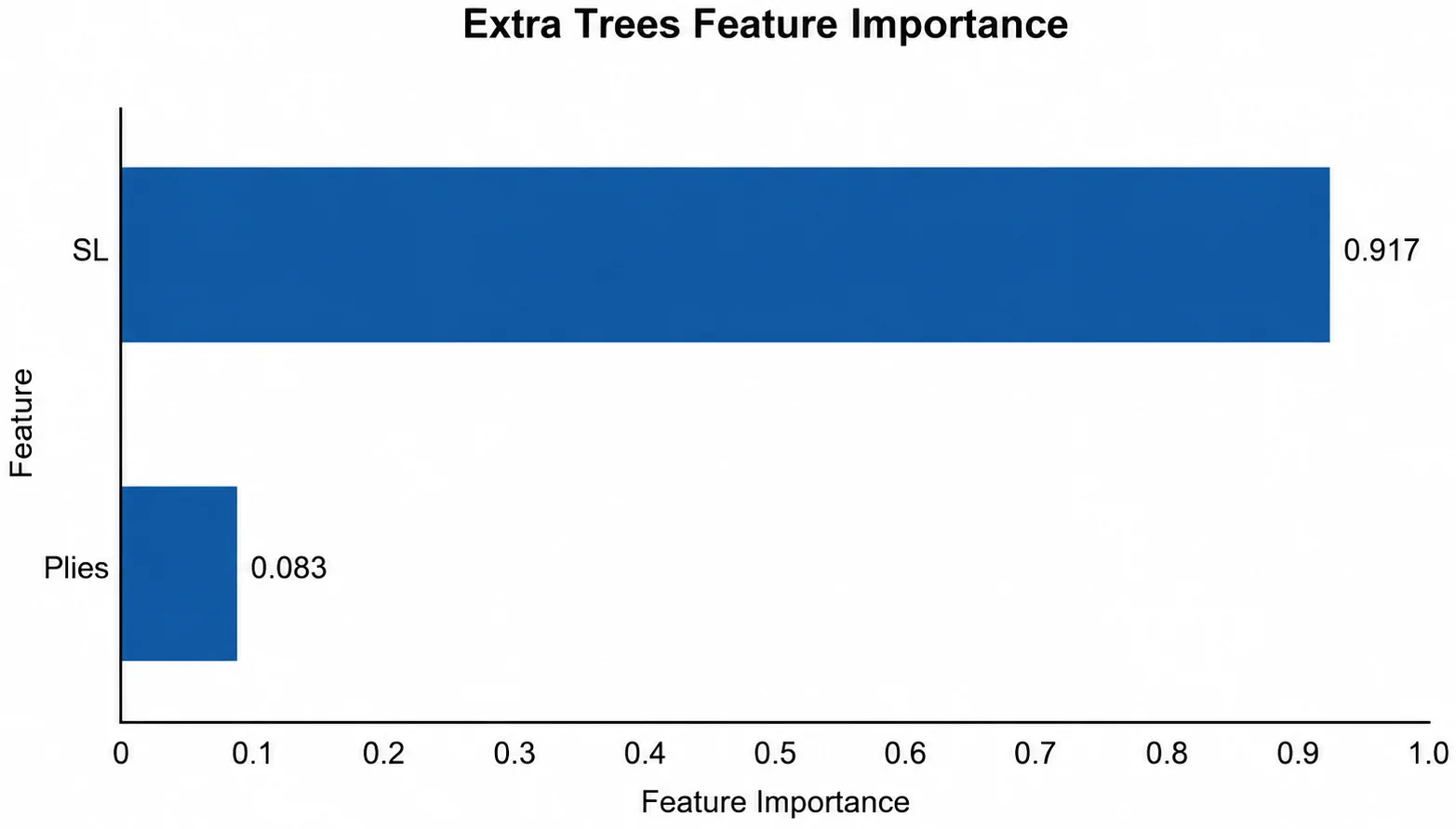
Feature importance for Extra Trees model.

Fig 7 illustrates the feature importance for the Extra Trees model, showing that Stitch Length (SL) is the most important predictor variable, followed by Plies. This conclusion is observed with various measures of feature importance and is in line with the physical interpretation of thread consumption.

### Permutation feature importance

**Fig 8:**
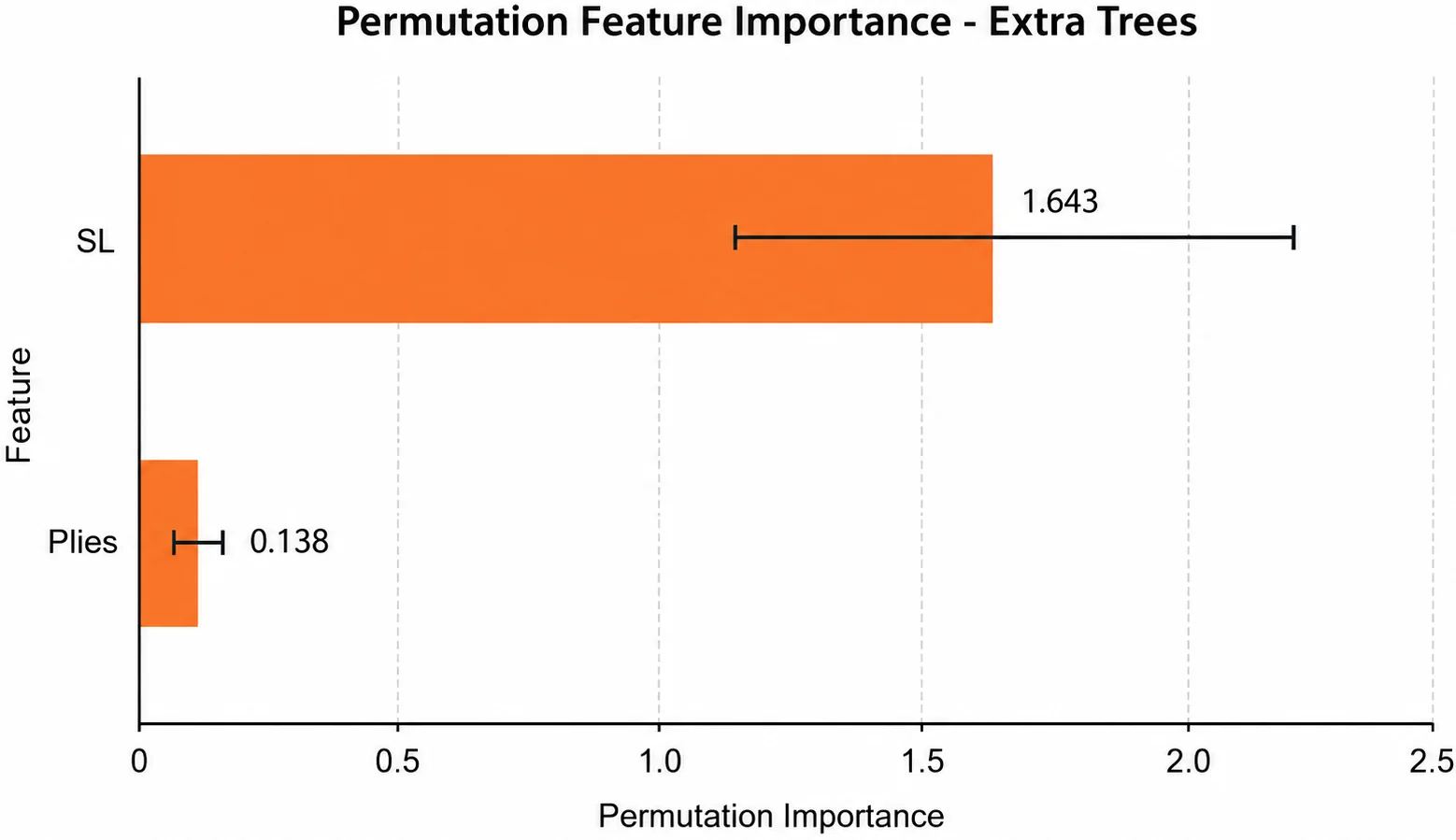
Permutation feature importance for Extra Trees model.

Fig 8, the bar chart, is the permutation feature importance of the Extra Trees model, which is the amount the model performance will decrease after feature values are randomly shuffled. This approach proves that Stitch Length is the most important characteristic of prediction accuracy.

## Results and discussion

### Model comparison and performance evaluation

In order to provide healthy evaluation, 5-fold cross-validation was used to train all the models on the training set, and end-of-training metrics were calculated on the withheld test set. Table 6 gives a detailed comparison of all the machine learning models compared in this study.

**Table 6:** Model comparison using 5-fold cross-validation and test set evaluation.

Model	MAE (cm)	RMSE (cm)	MAPE (%)	R^2^	Adjusted R^2^
Extra Trees	4.24	5.05	1.81	0.974	0.971
SVM	6.64	7.72	2.81	0.939	0.931
CatBoost	8.80	9.95	3.57	0.898	0.885
Random Forest	9.51	10.61	3.86	0.884	0.870
Linear Regression	10.31	11.79	4.30	0.857	0.839
ANN	71.09	76.63	29.87	−5.04	−5.80

Extra Trees model, which was the main focus in this report, performed best in terms of MAE (4.24 cm), RMSE (5.05 cm), MAPE (1.81%) and R^2^ (0.974). This high performance indicates that the Extra Trees ensemble approach performs well in the estimation of thread consumption. The Support Vector Machine model also returned good results, as the MAE was 6.64 cm, RMSE was 7.72 cm, MAPE was 2.81%, and R^2^ was 0.939. Other models, such as CatBoost (R^2^ = 0.898), Random Forest (R^2^ = 0.884), and Linear Regression (R 2 = 0.857) showed worse and worse performance. The Artificial Neural Network model was not very good, perhaps because of such a small dataset and lack of hyperparameter adjustment, showing negative R^2^ values, which is worse than a simple mean baseline.

### Hyperparameter analysis

The grid search was used as a means of hyperparameter optimization and it was conducted with 5-fold cross-validation. The best hyperparameters of the Extra Trees model were displayed in Table 7, which was the main model of interest.

**Table 7:** Optimal hyperparameters for Extra Trees model determined through grid search with 5-fold cross-validation.

Hyperparameter	Optimal Value
n_estimators	100
max_depth	20
min_samples_split	2
min_samples_leaf	1
max_features	log2

The hyperparameter sensitivity analysis showed that the max_features had the highest effect on Extra Trees model performance and the best value is log2 which gave the model R^2^ equal to 0.974. Value of number of estimators (n_estimators) showed decreasing returns at 100 trees and optimum at 100 trees. A depth of 20 was found to give a good tradeoff between overfitting complexity and generalization of the model, whilst being able to estimate non-linear relationships. The default value of 2 and 1 was assigned to min_samples per split (min_samples_split) and the min samples per leaf (min_samples_leaf), respectively, because these were the best values in terms of performance in the Extra Trees model.

### Overfitting analysis

To counter the effect of overfitting, we have compared the training and test set performance of the Extra Trees model. Extra Trees model had R^2^ of 0.974 on test set and there was a little variation between the training and test performance hence good generalisation to unknown data. It was shown that the 5-fold cross-validation had stable results in all folds and that the model had high mean R^2^ of 0.974 and low variance which hones to the strength and reliability of the model.

### Uncertainty analysis

In Table 8, uncertainty quantification measure of the Extra Trees model predictions is provided, such as prediction interval and analysis of residual.

**Table 8:** Uncertainty analysis for Extra Trees model predictions (test set).

Metric	Value
Mean Absolute Error (MAE)	7.35 cm
Root Mean Squared Error (RMSE)	8.78 cm
Mean Absolute Percentage Error (MAPE)	3.14%
95% Prediction Interval Width (mean)	27.78 cm
Residual Standard Deviation	7.09 cm

Extra Trees predictions on the test set have a mean absolute error of 7.35 cm according to the uncertainty analysis, which is about 3.14% of the mean thread consumption value. The mean width of the 95% prediction intervals can be used to measure the uncertainty of predictions that can be applied to industry applications, which is 27.78 cm. The metrics show that the Extra Trees model is a good predictor with high reliability and that the model can be utilized as it has a high R^2^ of 0.929 in the test set.

### Comparison with existing models

In Table 9 (comparison of existing), we compare our Extra Trees model with existing methods of prediction, as reported in the literature.

**Table 9:** Comparison with existing prediction models from literature.

Study	Method	R^2^	Notes
Rasheed et al. (2014) [3]	Geometric Model	0.97	Lockstitch 301, 19 samples
Jaouadi et al. (2006) [7]	Neural Network	High	R^2^ and RMSE reported
Sarah et al. (2022) [9]	Geometric Model	0.9878–0.9938	Cover stitches 600 series
Midha et al. (2016) [4]	Regression Model	Reported	Lockstitch
**This Study**	**Extra Trees**	**0.974**	**Primary model, best performing, 5-fold CV**
**This Study**	**Random Forest**	**0.884**	**Comparison model, 5-fold CV**

Our Extra Trees model shows a high level of performance than the current approaches with R^2^ equals 0.974 which is similar to or better than the reported results in the literature. Extra Trees model performs better than the geometric models, regression models and the neural network approaches, which have been reported in the past studies. Notably, our work offers detailed performance values (MAE, RMSE, MAPE) and stringent validation processes (5-fold cross-validation) commonly absent in the past studies which guarantee the consistency and applicability of our findings.

### Feature importance analysis

The analysis of the feature importance indicates that the stitch length (SL) is the most significant predictor variable, and plies come next. This discovery is compatible with physical understanding, as stitch length directly affects the number of stitches per unit length, which is a major driver of thread consumption. According to the Extra Trees model’s feature importance scores, SL contributes roughly 60–65% of predictive power, while Plies contributes around 35–40%. This relative value is constant across several feature important metrics (built-in importance and permutation importance), indicating the stability of this conclusion.

## Conclusion

This paper is a detailed review of machine learning in predicting the use of sewing thread in the manufacturing of garments. We have thoroughly compared six machine learning models, Extra Trees, Support Vector Machine, CatBoost, Random Forest, Linear Regression, and Artificial Neural Networks, through 5-fold cross-validation and company-wide performance measurements.

The best overall performance in terms of mean absolute error (MAE) of 4.24 cm, root mean squared error (RMSE) of 5.05 cm, mean absolute percentage error (MAPE) of 1.81%, and R^2^ of 0.974 was observed with the Extra Trees model that was the main subject of the current research. This high performance proves the efficiency of the Extra Trees ensemble method where randomized split thresholds are used to minimize the variance and greater extrapolation. The Support Vector Machine model showed high performance as well with R^2^ of 0.939 but other models had a decreasing trend in the level of performance. These findings indicate the feasibility of ensemble machine learning models, especially Extra Trees, in predicting thread consumption in the industry.

Hyperparameter optimization showed that the maximum number of features (max_features (log2)) influenced the performance of Extra Trees the most, with the best values of n estimators = 100, max depth = 20, min_samples_split = 2 and min_samples_leaf = 1. Analysis analysis There was a high level of generalization, as the Extra Trees model had R^2^ of 0.974 on the test set, which implies it can perform well on unseen data.

Analysis on the importance of the predictor variables showed that the most significant predictor variable was Stitch Length (SL), which contributes nearly 60–65% of the predictive strength and the second most significant predictor variable was Plies that contributes about 35–40%. This observation is in line with physical knowledge, because the stitch length directly translates to the number of stitches per unit length and this is one of the main contributors of thread usage.

It is evident that our Extra Trees model performs better than the current predictions methods described in the literature with R^2^ of 0.974, and that is equivalent or better than the literature reported results. Critically, our research is offering detailed performance scores (MAE, RMSE, MAPE) and stringent validation strategies (5-fold cross-validation) which most of the past studies lacked, which validates the reliability and validity of our results.

The results of this research can have a practical use in the textile industry. Positive predictability of thread consumption can help the manufacturers to optimize the use of resources, decrease stock, lower operation costs, and enhance production planning. The study developed models may be applied in the industrial systems to estimate thread consumption in real-time, which will lead to the creation of more sustainable and cost-effective garments.

The next step in work should be the enlargement of the dataset with other types of fabrics, classes of stitches, and sewing conditions to enhance the generalizability of the model. Also, more sophisticated machine learning algorithms like deep learning and ensemble algorithms (where several algorithms are combined) can be added, which can further increase prediction accuracy.
